# Gene master regulators of papillary and anaplastic thyroid cancers

**DOI:** 10.18632/oncotarget.23417

**Published:** 2017-12-19

**Authors:** Dumitru A. Iacobas, Neha Y. Tuli, Sanda Iacobas, John K. Rasamny, Augustine Moscatello, Jan Geliebter, Raj K. Tiwari

**Affiliations:** ^1^ Department of Pathology, New York Medical College, Valhalla, NY, USA; ^2^ Department of Microbiology & Immunology, New York Medical College, Valhalla, NY, USA; ^3^ Department of Otolaryngology, New York Medical College, Valhalla, NY, USA; ^4^ Center for Computational Systems Biology at Prairie View A&M University, Prairie View, TX, USA

**Keywords:** 8505C anaplastic thyroid cancer cell line, BCPAP papillary thyroid cancer cell line, DDX19B, NEMP1, PANK2

## Abstract

We hypothesize that distinct cell phenotypes are governed by different sets of gene master regulators (GMRs) whose strongly protected (by the homeostatic mechanisms) abundance modulates most cell processes by coordinating the expression of numerous genes from the corresponding functional pathways. Gene Commanding Height (GCH), a composite measure of gene expression control and coordination, is introduced to establish the gene hierarchy in each phenotype. If the hypothesis is true, than one can selectively destroy cancer nodules from a heterogeneous tissue by altering the expression of genes whose GCHs are high in cancer but low in normal cell phenotype. Here, we test the hypothesis and show its utility for the thyroid cancer (TC) gene therapy. First, we prove that malignant and cancer free surrounding areas of a surgically removed papillary TC (PTC) tumor are governed by different GMRs. Second, we show that stable transfection of a gene induces larger transcriptomic alterations in the cells where it has higher GCH than in other cells. For this, we profiled the transcriptomes of the papillary BCPAP and anaplastic 8505C TC cell lines before and after stable transfection with NEMP1, DDX19B, PANK2 or UBALD1. The four genes were selected to have similar expression levels but significantly different GCH scores in the two cell lines before transfection. Indeed, each of the four genes triggered larger alterations in the cells where they had larger GCH. Our results prove the feasibility of a personalized gene therapy approach that selectively targets the cancer cells from a tissue.

## INTRODUCTION

Thyroid cancer (TC) is the most common endocrine malignancy in United States with rapidly rising incidence. The American Cancer Society estimates 56,850 new cases of thyroid cancer in 2017. Morphologically, the thyroid cancers are classified as papillary, follicular, medullary and anaplastic. Differentiated papillary (PTC) and follicular forms comprise 90-95% of thyroid cancers and are treatable, whereas anaplastic thyroid cancer (ATC) is the rarest but the most fatal and incurable form of the disease, with a median survival of 5 months [1]. ATC is an undifferentiated cancer arising presumably from pluripotent thyroid progenitor cells and accumulating genetic lesions. ATC is aneuploid with several chromosomal abnormalities and loss of heterozygosity. ATC can range from poorly differentiated TC characterized by high mitotic index to squamaous TC where its thyroid origin is difficult to ascertain.

For most ATCs, mutation of BRAF (v-raf murine sarcoma viral oncogene homolog B) or in rare cases, detection of RET/PTC and PAX8/PPAR fusions suggests thyroid origin of cells [2–4]. Presence of the tumor protein TP53 and β-catenin commonly found in ATCs but occasionally seen in poorly differentiated carcinoma also suggests tumor dedifferentiation [5–10]. ATC etiology is not completely understood; however several premises indicate that it originates from thyroid stem cells to manifest an undifferentiated, but aggressive phenotype [11, 12]. It is believed that both the papillary and follicular thyroid cancers result from a similar differentiation program coupled with the development of mutations most notably RET/PTC, RAS and BRAF for PTC and/or PAX8/PPARΎ [13–17]. However, genetic lesions are not uniformly distributed and no single or group of mutations define ATC [12, 13, 18–20]. The heterogeneity of tumor cell differentiation and the repertoire of genetic lesions (although TC has the least numbers of genetic lesions as compared to other TCs) makes difficult the task of developing a universal therapy with demonstrated clinical efficacy [21, 22].

There are several interactive open-access dbases of cancer transcriptomic signatures and even an Atlas of the Human Cancer Transcriptome [23] presenting favorable and unfavorable prognostic genes with associated Kaplan-Mayer surviving diagrams. In previous papers [24–28], we have analyzed the significance and utility of several potential TC biomarkers and therapeutic targets and their dependence on sex hormones [29–32]. However, being selected from the most frequently altered (as sequence or/and expression) genes in large population cohorts, the biomarkers appeared as less protected by the homeostatic mechanisms like low players in cell life. Therefore, restoration of the structure/expression of the altered biomarkers has most likely little therapeutic value. This explains why so far, no TC gene biomarker [33, 34] proved therapeutically efficient. Moreover, not only the biomarker(s) but thousands other genes are altered in TC, in (although partially overlapping) never repeatable combinations and nobody knows whether the neglected contributions of the other gene alterations are really negligible.

If cancer and normal cells are governed by distinct gene master regulators (GMRs) than “smart” manipulation of GMRs would selectively destroy the cancer nodules without much damage to the surrounding healthy tissue. The idea of master regulators has been around for almost four decades, most authors looking for transcription factors occupying the top of the regulatory hierarchy that determines cell fate and differentiation. Sophisticated algorithms using reverse engineering of transcriptional networks have been proposed and validated for use in therapeutic decision making [35]. KEGG (http://www.genome.jp), GenMapp (http://genmapp.org), IPA (http://ingenuity.com), DAVID (http://david.abcc.ncifcrf.gov) and other popular software have been developed to ensemble the biomarkers and other genes in functional pathways. Regardless of the method (Pearson correlation, Boolean, Bayesian, differential equations or just knowledge-based) to network the genes with respect to their co-regulation in different conditions [36, 37], such algorithms implicitly assumes ironclad functional pathways. This is a major weakness given the evident morphological and physiological changes during cancerization and in response to chemo-, radio and cell therapy. Although manually curated by genomic experts, the functional pathways constructed by these (actually) text miners are also “too” universal, lacking specificity with respect to race/strain, sex, age, and risk factors. Moreover, they are deterministic (unique network) in spite of the stochastic nature of the chemical reactions leading to an environmentally depending spectrum of possible “wirings” of the same subset of genes.

We consider as GMR a gene whose highly protected expression by the homeostatic mechanisms governs the phenotype by regulating the transcription of genes involved in major functional pathways through expression coordination. The high protection (indicating the critical importance for cell life) confines expression oscillations of GMRs in narrow intervals. Therefore, GMRs are rarely found spontaneously regulated and by consequence not selected as biomarkers. We estimate the protection of GMR from the reduced expression variability and its power to modulate a pathway from the Pearson correlation with expression oscillations of the pathway genes in biological replicas (obtained by splitting in four the malign and normal regions of the removed tumor).

Our approach, consistent with the Genomic Fabric Paradigm (GFP, [38, 39], does not provide novel biomarkers for TC diagnostic in ALL patients but a revolutionary way to cure the TC of the ACTUAL patient. The genomic fabric is defined as the transcriptome associated with the most interconnected and stably expressed network of genes responsible for a particular functional pathway. The fabric exhibits specificity with respect to race/strain, sex and sex hormones, age, tissue/cell type, and life style and environmental factors. It remodels during development, progression of a disease and in response to external stimuli and treatments.

Recently [40], we tested the hypothesis that *cancer and normal cells are governed by different GMRs* in the normal cortex and two primary tumor regions of a surgically removed clear cell renal cell carcinoma from a 74y old male. Here, we test again this hypothesis in a papillary cancer and cancer free surrounding tissue from the left thyroid lobectomy of a 33y old female. The efficacy of GMR targeting was checked by determining the transcriptomic effects of stable transfection of genes with different GCHs in the papillary BCPAP [41] and anaplastic 8505C [42] human TC cells that we used in previous studies [25, 27–29].

## RESULTS

Experimental methods and raw and normalized gene expression data complying with the “Minimum Information about Microarray Experiments” (MIAME, [43]) were deposited in https://www.ncbi.nlm.nih.gov/gds/ and are publically accessible as GSE97001, GSE97002, GSE97028, GSE97030, GSE97031 and GSE97427.

### Cancer alters expression and coordination of numerous genes

**Figure 1:**
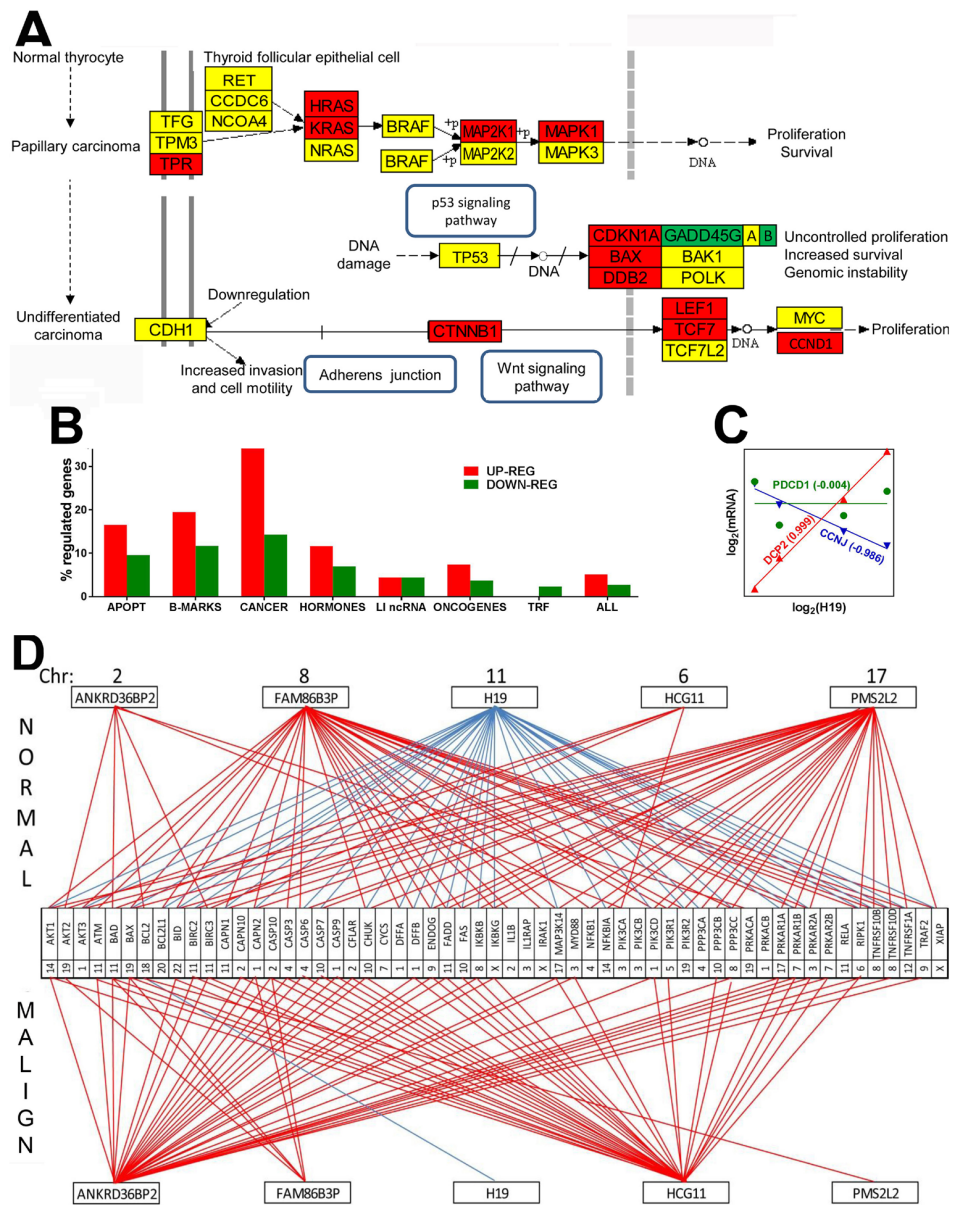
Papillary cancer of the thyroid regulates numerous genes and remodel transcriptomic networks **(A)** Regulation of the KEGG-determined pathway of thyroid cancer. Red/green/yellow background of the gene symbol indicates up-/down/no regulation. GADD45G|A|B indicates the three growth arrest and DNA-damage-inducible genes: gamma, alpha and beta. **(B)** Percentages of up- and down regulated genes in various groups. APOPT = apoptosis, B-MARKS = biomarkers, CANCER = thyroid cancer, HORMONES = thyroid hormones, LI ncRNAs = long intergenic non-protein coding RNAs, TRF = transcription factors, ALL = the entire transcriptome. **(C)** Examples of genes synergistically (*DCPP2* = decapping mRNA 2), antagonistically (*CCNJ =* cyclin J) and independently (*PDCD1* = programmed cell death 1) expressed with H19 (= H19, imprinted maternally expressed transcript, long non-coding RNA). Numbers near gene symbols are Pearson pair-wise correlation coefficients between the (log_2_) expression levels of *H19* and linked gene. **(D)** Example of remodeling of the transcriptomic networks by which ncRNAs regulate apoptosis. Red/blue line indicates significant (p-val < 0.05) synergism/antagonism between the linked gene and ncRNA. Numbers close to genes and ncRNAs’ symbols are their hosting chromosomes (Chr).

Compared to the normal tissue, expression of 5.1%% of the quantified unigenes was up-regulated and of 2.7% was down-regulated in the malignant region of the excised thyroid tumor. When comparison was extended to the papillary BCPAP cells, 17.10% of the genes were up-regulated and 15.53 down-regulated, while in the anaplastic 8505C cells, 17.09% of the genes were up- and 18.79% were down-regulated. Table 1 lists the fold-change (negative for down-regulation) and the p-value of the regulation, and Figure 1A the interconnection of the genes included in the KEGG^1^ -determined pathway of thyroid cancer with respect to the surrounding unaffected tissue of thyroid.

**Table 1:** Regulation of the genes included in the KEGG-determined functional pathway of thyroid cancer

Gene	Systematic name	Description	FC	p-val
BAK1	NM_001188	BCL2-antagonist/killer 1	1.05	0.9125
BAX	NM_138764	BCL2-associated X protein	2.18	0.0190
BRAF	NM_004333	v-raf murine sarcoma viral oncogene homolog B	1.52	0.3870
CCDC6	NM_005436	coiled-coil domain containing 6	-1.28	0.5853
CCND1	NM_053056	cyclin D1	2.90	0.0223
CDH1	NM_004360	cadherin 1, type 1, E-cadherin	1.17	0.8237
CDKN1A	NM_078467	cyclin-dependent kinase inhibitor 1A	2.65	0.0205
CTNNB1	NM_001904	catenin	2.59	0.0191
DDB2	NM_000107	damage-specific DNA binding protein 2, 48kDa	2.31	0.0603
GADD45A	NM_001924	growth arrest and DNA-damage-inducible, alpha	-1.52	0.3406
GADD45B	NM_015675	growth arrest and DNA-damage-inducible, beta	-2.07	0.0895
GADD45G	NM_006705	growth arrest and DNA-damage-inducible, gamma	-2.00	0.0222
HRAS	NM_005343	Harvey rat sarcoma viral oncogene homolog	1.80	0.0254
KRAS	NM_004985	Kirsten rat sarcoma viral oncogene homolog	2.84	0.0208
LEF1	NM_016269	lymphoid enhancer-binding factor 1	1.91	0.0323
MAP2K1	NM_002755	mitogen-activated protein kinase kinase 1	1.71	0.0150
MAP2K2	NM_030662	mitogen-activated protein kinase kinase 2	1.35	0.5240
MAPK1	NM_138957	mitogen-activated protein kinase 1	1.88	0.0308
MAPK3	NM_002746	mitogen-activated protein kinase 3	1.28	0.6278
MYC	NM_002467	v-myc avian myelocytomatosis viral oncogene homolog	-1.46	0.4439
NCOA4	NM_001145260	nuclear receptor coactivator 4	-1.27	0.5110
NRAS	NM_002524	neuroblastoma RAS viral	-1.26	0.6097
NTRK1	NM_002529	neurotrophic tyrosine kinase, receptor, type 1	***not available***	
PAX8	NM_003466	paired box 8	-1.99	0.0174
POLK	NM_016218	polymerase	-1.10	0.8021
PPARG	NM_138711	peroxisome proliferator-activated receptor gamma	-5.00	0.0911
RET	NM_020630	ret proto-oncogene	1.10	0.8589
RXRA	NM_002957	retinoid X receptor, alpha	-1.66	0.2898
RXRB	NM_021976	retinoid X receptor, beta	-1.09	0.8583
RXRG	NR_033824	retinoid X receptor, gamma	***not available***	
TCF7	NM_003202	transcription factor 7	4.26	0.0724
TCF7L1	NM_031283	transcription factor 7-like 1	-4.08	0.0492
TCF7L2	NM_001198531	transcription factor 7-like 2	-1.02	0.9648
TFG	NM_006070	TRK-fused gene	-1.28	0.5636
TP53	NM_001126118	tumor protein p53	1.37	0.1237
TPM3	NM_001043352	tropomyosin 3	1.34	0.3639
TPR	NM_003292	translocated promoter region, nuclear basket protein	2.51	0.0196

Gene expression levels in the cancer nodule were compared with the corresponding values in the surrounding unaffected tissue of the profiled thyroid.Red/green/yellow background in the original tables the gene symbols had red/green/yellow background. Please add the background. For instance, the last gene symbol TPR should be in a red background of the gene symbol indicates up-/down-/no regulation, will white background indicates that the expression of the corresponding gene was not quantifiable in all 4 samples from the cancer nodule AND all 4 samples from the unaffected surrounding tissue. FC = fold-change (negative for down-regulation), p-val = p-value of the heteroscedastic *t*-test of the equality of the average expression level in the tumor side and in the surrounding normal tissue.

We found a significant overexpression of members of the *Ras* oncogene family *HRAS* and *KRAS* (known for their prominent roles in various types of cancer (e.g. [44, 45]. MAP2K1 (mitogen-activated kinase 1), part of the RAS/MAPK pathways, which informs the cell nucleus about the extracellular chemical environment was also up-regulated. Expression of *TP53* was not affected, indicating lack of ATC development [46, 47]. Figure 1B presents the percentages of up- and down regulated genes in several well-defined groups of genes. Interestingly, out of the investigated groups of genes, the biomarkers selected from [48] formed the second most regulated group (after the thyroid cancer genes), confirming their value for TC diagnosis. In contrast, the transcription factors (TRF) formed the least regulated group (0.0% up and 2.3% down). With 16.5% up and 9.6% down-regulated genes (listed in Table 2) apoptosis is also a major pathway with substantial alteration in thyroid cancer.

**Table 2:** Significantly regulated apoptosis genes in the cancer nodule with respect to the surrounding unaffected tissue of the profiled thyroid

Gene	Systematic name	Description	FC	p-val
BAX	NM_138764	BCL2-associated X protein	2.18	0.0190
BBC3	NM_014417	BCL2 binding component 3	2.80	0.0073
BCL2	NM_000633	B-cell CLL/lymphoma 2	-3.34	0.0087
BCL2L11	NM_138621	BCL2-like 11	-3.25	0.0073
BID	NM_197966	BH3 interacting domain death agonist	5.77	0.0317
BIRC5	NM_001012271	baculoviral IAP repeat containing 5	4.14	0.0090
CASP3	NM_004346	caspase 3, apoptosis-related cysteine peptidase	4.01	0.0379
CTSH	NM_004390	cathepsin H	6.39	0.0044
CTSK	NM_000396	cathepsin K	-1.88	0.0126
CTSV	NM_001333	cathepsin V	-1.80	0.0282
CYCS	NM_018947	cytochrome c, somatic	-2.14	0.0182
DAB2IP	NM_032552	DAB2 interacting protein	2.88	0.0099
FAS	NM_000043	Fas cell surface death receptor	2.79	0.0075
FOS	NM_005252	FBJ murine osteosarcoma viral oncogene homolog	-2.15	0.0140
GADD45B	NM_015675	growth arrest and DNA-damage-inducible, beta	-2.07	0.0089
GADD45G	NM_006705	growth arrest and DNA-damage-inducible, gamma	-2.00	0.0222
GZMB	NM_004131	granzyme B	-2.27	0.0081
HRAS	NM_005343	Harvey rat sarcoma viral oncogene homolog	1.80	0.0254
HRK	ENST00000257572	harakiri, BCL2 interacting protein	3.01	0.0065
ITPR2	NM_002223	inositol 1,4,5-trisphosphate receptor, type 2	3.28	0.0079
JUN	NM_002228	jun proto-oncogene	-3.38	0.0055
KRAS	NM_004985	Kirsten rat sarcoma viral oncogene homolog	2.84	0.0208
LMNA	NM_005572	lamin A/C	2.92	0.0053
MAP2K1	NM_002755	mitogen-activated protein kinase kinase 1	1.71	0.0150
MAPK1	NM_138957	mitogen-activated protein kinase 1	1.88	0.0308
NFKB1	NM_003998	nuclear factor of kappa light polypeptide gene enhancer in B-cells 1	2.15	0.0069
PARP1	NM_001618	poly (ADP-ribose) polymerase 1	2.50	0.0078
TNFRSF10B	NM_003842	tumor necrosis factor receptor superfamily, member 10b	1.84	0.0080
TNFRSF10C	NM_003841	tumor necrosis factor receptor superfamily, member 10c, decoy without an intracellular domain	7.14	0.0242
TNFSF10	NM_003810	tumor necrosis factor (ligand) superfamily, member 10	-4.11	0.0064

Red/green background of the gene symbol indicates significant up-/down regulation. FC = fold-change (negative for down-regulation), p-val = p-value of the heteroscedastic *t*-test of the equality of the average expression level in the tumor side and in the surrounding normal tissue.

We found also substantial remodeling of the transcriptomic networks by which the long intergenic non-protein coding RNAs (LI ncRNA) modulate major functional pathways through expression coordination (principle in Figure 1C) with pathway genes. Thus, Figure 1D presents the remodeling of part of the transcriptomic networks that LI ncRNAs form with apoptotic genes (coordination values in Table 3). Notably, some synergistically expressed gene pairs in normal tissue became independently expressed in cancer (*ANKRD36BP2-BCL2*, *PMS2L2-TNFRSF10D, PMS2L2-TNFRSF1A*) or an independently expressed pair (*HCG11-PPP3CB*) in normal became antagonistically expressed in cancer. *H19* strong antagonism with expression of apoptotic genes in normal tissue is practically cancelled in cancer, confirming its important role in cancer proliferation revealed by several authors [49]. Our coordination analysis revealed that LI ncRNAs can modulate expression of genes located not only on the same but also on other chromosomes. For instance, in the normal tissue, H19 from Chr 11 antagonizes apoptosis genes from Chr 1 (*AKT3, CAPN2, DFFA, DFFB, PIK3CD*), Chr 3 (*PRKAR2A*), Chr 4 (*CASP6, PPP3CA*), Chr 6 (*RIPK1*), Chr 7 (*PRKAR1B*). Chr 8 (*IKBKB, PPP3CC, TNFRSF10B TNFRSF10D*), Chr 9 (*ENDOG*), Chr 10 (*CASP7, CHUCK, FAS*), Chr 11 (*BIRC2, CAPN1, FADD*), Chr 12 (*TNFRSF1A*). Chr 14 (*AKT1, NFKBIA*), Chr 17 (*MAPK3K14, PRKAR1A*), Chr 19 (*BAX, PIK3R2*), Chr 20 (BCL2L1) and Chr X (*XIAP*). In the malign region, the (p < 0.05) significant expression coordination of *H19* is limited to a single apoptosis gene, *BCL2* (Chr 18) that, interestingly, is not coordinately expressed with *H19* in normal tissue. Supplementary Table 1 lists all regulated genes in the cancer nodule with respect to the surrounding normal tissue.

**Table 3:** Expression coordination values between LI ncRNA and apoptosis genes in normal and malign part of the tumor

Normal	ANKRD36BP2	FAM86B3P	H19	HCG11	PMS2L2	Malign	ANKRD36BP2	FAM86B3P	H19	HCG11	PMS2L2
AKT1	0.680	0.978	-0.908	0.649	0.929	AKT1	0.984	0.749	0.144	0.964	-0.173
AKT2	0.940	0.693	-0.519	0.948	0.591	AKT2	0.858	0.999	0.205	0.834	-0.180
AKT3	0.361	0.983	-0.999	0.313	0.986	AKT3	0.672	0.357	-0.446	0.590	0.399
ATM	0.786	0.934	-0.835	0.756	0.858	ATM	0.953	0.644	0.356	0.967	-0.394
BAD	0.982	0.610	-0.421	0.986	0.483	BAD	0.911	0.952	0.451	0.923	-0.438
BAX	0.599	0.994	-0.945	0.567	0.963	BAX	0.951	0.950	0.122	0.918	-0.118
BCL2	0.921	0.793	-0.652	0.888	0.667	BCL2	0.008	-0.120	-0.939	-0.117	0.913
BCL2L1	0.574	0.952	-0.922	0.509	0.888	BCL2L1	0.794	0.371	-0.074	0.763	0.015
BID	0.362	0.958	-0.979	0.297	0.940	BID	0.993	0.886	0.314	0.989	-0.324
BIRC2	0.099	0.892	-0.952	0.075	0.962	BIRC2	0.942	0.708	0.533	0.978	-0.559
BIRC3	0.981	0.670	-0.494	0.968	0.534	BIRC3	0.952	0.810	0.538	0.983	-0.551
CAPN1	0.195	0.938	-0.991	0.144	0.966	CAPN1	0.808	0.359	0.099	0.801	-0.160
CAPN10	0.871	0.862	-0.730	0.855	0.772	CAPN10	0.994	0.780	0.195	0.979	-0.221
CAPN2	0.332	0.979	-1.000	0.287	0.989	CAPN2	0.920	0.978	0.143	0.888	-0.130
CASP10	0.302	0.809	-0.846	0.210	0.757	CASP10	0.949	0.952	0.122	0.916	-0.118
CASP3	0.806	0.922	-0.816	0.778	0.842	CASP3	0.963	0.868	0.482	0.984	-0.488
CASP6	0.547	0.994	-0.955	0.519	0.978	CASP6	0.932	0.593	0.122	0.917	-0.167
CASP7	0.091	0.899	-0.968	0.055	0.960	CASP7	0.968	0.826	0.490	0.991	-0.503
CASP9	-0.073	0.727	-0.799	-0.065	0.846	CASP9	0.986	0.837	0.410	0.998	-0.425
CFLAR	0.880	0.843	-0.718	0.841	0.726	CFLAR	0.980	0.712	0.283	0.980	-0.316
CHUK	0.327	0.976	-0.992	0.293	0.999	CHUK	0.988	0.753	0.320	0.992	-0.348
CYCS	0.059	0.773	-0.814	0.073	0.875	CYCS	0.793	0.655	0.792	0.868	-0.802
DFFA	0.648	0.954	-0.903	0.590	0.881	DFFA	0.763	0.349	-0.167	0.720	0.108
DFFB	0.503	0.916	-0.905	0.428	0.851	DFFB	0.981	0.720	0.181	0.967	-0.214
ENDOG	0.485	0.995	-0.973	0.455	0.991	ENDOG	0.981	0.887	0.399	0.989	-0.406
FADD	0.691	0.975	-0.903	0.659	0.922	FADD	0.988	0.745	0.207	0.976	-0.237
FAS	0.418	0.993	-0.994	0.373	0.987	FAS	0.871	0.742	0.697	0.928	-0.707
IKBKB	0.089	0.873	-0.930	0.073	0.952	IKBKB	0.930	0.899	0.523	0.954	-0.520
IKBKG	0.377	0.973	-0.989	0.319	0.960	IKBKG	0.999	0.814	0.251	0.990	-0.272
IL1B	0.602	0.918	-0.881	0.531	0.838	IL1B	0.810	0.900	0.618	0.848	-0.597
IL1RAP	-0.476	0.427	-0.571	-0.466	0.595	IL1RAP	0.725	0.316	-0.239	0.674	0.181
IRAK1	0.683	0.919	-0.860	0.620	0.829	IRAK1	0.857	0.464	-0.003	0.831	-0.051
MAP3K14	0.342	0.970	-0.994	0.285	0.967	MAP3K14	0.993	0.767	0.254	0.987	-0.281
MYD88	0.865	0.856	-0.722	0.855	0.772	MYD88	0.982	0.868	0.415	0.993	-0.425
NFKB1	0.372	0.915	-0.899	0.374	0.956	NFKB1	0.647	0.870	0.620	0.687	-0.580
NFKBIA	-0.048	0.830	-0.925	-0.083	0.912	NFKBIA	0.665	0.748	0.808	0.737	-0.787
PIK3CA	0.957	0.544	-0.348	0.977	0.431	PIK3CA	0.753	0.283	0.018	0.739	-0.083
PIK3CB	0.706	0.866	-0.760	0.718	0.839	PIK3CB	0.723	0.226	0.206	0.738	-0.273
PIK3CD	0.566	0.998	-0.960	0.529	0.970	PIK3CD	0.983	0.769	0.403	0.997	-0.427
PIK3R1	0.423	0.907	-0.875	0.429	0.940	PIK3R1	0.893	0.505	0.311	0.908	-0.361
PIK3R2	0.328	0.974	-0.998	0.276	0.978	PIK3R2	0.876	0.549	-0.110	0.831	0.065
PPP3CA	0.106	0.872	-0.922	0.094	0.950	PPP3CA	0.788	0.329	0.077	0.779	-0.140
PPP3CB	0.049	0.829	-0.886	0.044	0.921	PPP3CB	0.880	0.579	0.592	0.930	-0.627
PPP3CC	0.512	1.000	-0.977	0.470	0.978	PPP3CC	0.949	0.621	0.232	0.947	-0.274
PRKACA	0.912	0.778	-0.641	0.872	0.644	PRKACA	0.749	0.316	-0.143	0.710	0.082
PRKACB	0.649	0.764	-0.709	0.566	0.641	PRKACB	0.680	0.238	-0.227	0.635	0.163
PRKAR1A	0.109	0.865	-0.913	0.100	0.946	PRKAR1A	0.935	0.671	0.520	0.971	-0.550
PRKAR1B	0.491	0.994	-0.980	0.441	0.968	PRKAR1B	0.984	0.733	0.188	0.970	-0.219
PRKAR2A	0.442	0.993	-0.981	0.411	0.996	PRKAR2A	0.982	0.771	0.408	0.997	-0.432
PRKAR2B	0.450	0.856	-0.858	0.364	0.785	PRKAR2B	0.968	0.672	0.224	0.962	-0.262
RELA	-0.152	0.750	-0.876	-0.213	0.818	RELA	0.805	0.382	-0.050	0.776	-0.009
RIPK1	0.433	0.994	-0.992	0.387	0.984	RIPK1	0.989	0.792	0.126	0.964	-0.150
TNFRSF10B	0.302	0.945	-0.956	0.287	0.988	TNFRSF10B	0.725	0.782	0.775	0.791	-0.758
TNFRSF10D	0.568	0.994	-0.952	0.539	0.973	TNFRSF10D	0.935	0.896	-0.038	0.882	0.036
TNFRSF1A	0.562	0.984	-0.954	0.508	0.937	TNFRSF1A	0.764	0.317	-0.063	0.737	0.001
TRAF2	-0.186	0.737	-0.867	-0.241	0.817	TRAF2	0.927	0.676	-0.079	0.880	0.045
XIAP	0.409	0.990	-0.995	0.361	0.984	XIAP	0.505	0.228	-0.630	0.405	0.587

Red/blue/yellow background of the coordination value indicates that the paired genes were significantly (p-val < 0.05) synergistically/antagonistically/ independently expressed in the indicated region. Note the differences between the two regions.

### Cancer and normal cells have different gene hierarchies in the thyroid

**Figure 2:**
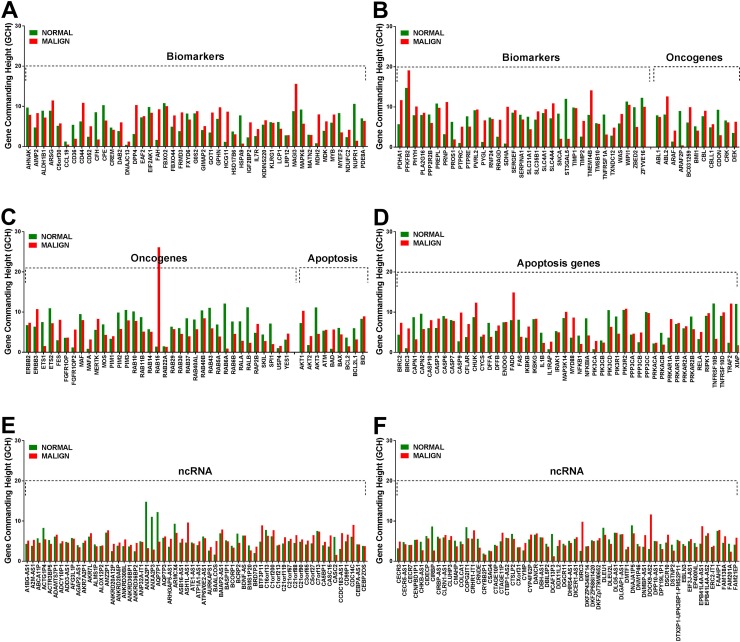
Gene commanding height (GCH) of selected TC biomarkers **(A, B)** oncogenes (B, C) apoptosis genes **(D)** and non-coding RNAs **(E, F)** in the normal and malign part of the surgically removed TC tumor. Note the differences between the GCH scores in the two regions.

Like our previous finding in a case of clear cell renal cell carcinoma [40] the microarray experiment on the left thyroid lobectomy proved existence of genes with large GCH differences between the cancer and the normal part of the tumor. Figure 2 presents the gene commanding heights (GCH) in normal and malign regions of the analyzed thyroid tumor for several TC biomarkers, oncogenes, apoptosis genes and ncRNAs. Note that no biomarker has high GCH, explaining why none of them proved therapeutically efficient for TC. However, with GCH = 26.14 in the malign part and 1.41 (18.5x smaller) in the normal tissue, the 2.71x significantly up-regulated member of the RAS oncogene family *RAB15* may be therapeutically actionable for this person as reported for other cancer cases (e.g. [50]).

### Anaplastic and papillary TC phenotypes have major differences in cell-cycle pathway and gene networking

**Figure 3:**
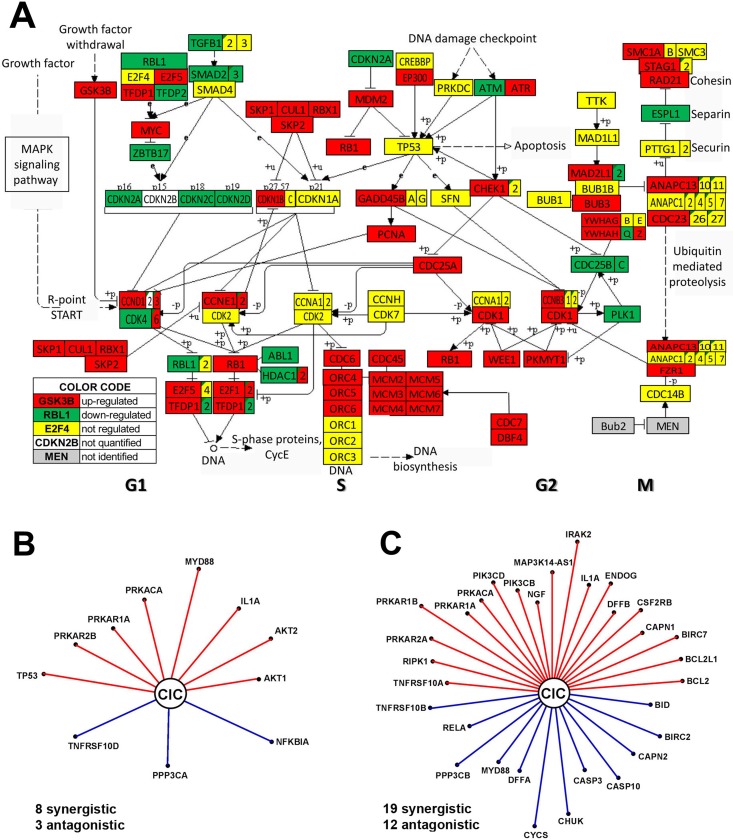
Major differences between anaplastic 8505C and papillary BCPAP TC cells include expression of cell-cycle pathway genes **(A)** and transcriptomic networks by which *CIC* (= capicua transcriptional repressor) coordinates expression of apoptotic genes **(B–C)**. In B and C a red/blue line indicates that *CIC* and the other gene are (p-val < 0.1) synergistically/antagonistically expressed. In order to visualize the strength of the expression coordination, the length of the line is proportional to ρ^3^ (ρ = Pearson pair-wise correlation coefficient). Thus, longer distances to *CIC* (as for *PRKAR1B, IRAK2* and *CYCS* in the 8505C network) indicate stronger expression coordination and by consequence stronger downstream influence. Note the differences between the two networks.

The transcriptomic profiles of the anaplastic (8505C) and papillary (BCPAP) TC cell lines were largely different, with the largest differences (61% = 45% up- and 16% down) in the expression of cell-cycle pathway genes (Figure 3A). This finding explains the accelerated progression to the undifferentiated form of the TC in 8505C cells [51]. Significant expression differences were noticed in both DNA replication (S phase) and mitosis (M phase) phases of the mitotic cell cycle progression as well as in the temporal gaps known as G1 and G2 phases. Such differences between the two TC cell lines justify some of the therapeutic approaches of the ATC. Thus, overexpression of the key regulatory cyclin-dependent kinase *CDK1* explains the choice of dinaciclib, a cyclin-dependent kinase inhibitor [52], while overexpression of the proto-oncogene *MYC* justifies the use of MYC potent inhibitor JQ1 [53]. *CDK6* is overexpressed and *CDK4* under-expressed, while *CDK2* and *CDK7* were similarly expressed in the two TC cell lines. All mini-chromosome maintenance genes (*MCM2, MCM3, MCM4, MCM5, MCM6, MCM7*), required to initiate eukaryotic DNA replication, were overexpressed in the 8505C cells, confirming previous reports (e.g. [54]) on other human ATC cell lines. Also overexpressed are three members of the origin recognition complex (*ORC4, ORC5, ORC6*) that were also previously linked to cancer development (e.g. [55]).

The transcriptomes of the two phenotypes also differ in the way the genes are networked to accomplish various biological processes. For instance, Figure 3B and 3C present the apoptosis genes that are significantly (p < 0.05) coordinately expressed with *CIC* (= capicua transcriptional repressor) a gene related to the various forms of cancer (e.g.: [56, 57]. Of note is that *CIC* coordinates the expression of many more apoptosis genes in the anaplastic phenotype and that only synergism with *IL1A, PRKACA* and *PRKAR1A* is common to both phenotypes.

### Predictive value of GCH score: expression manipulation of a gene has larger effects on cells it commands

**Figure 4:**
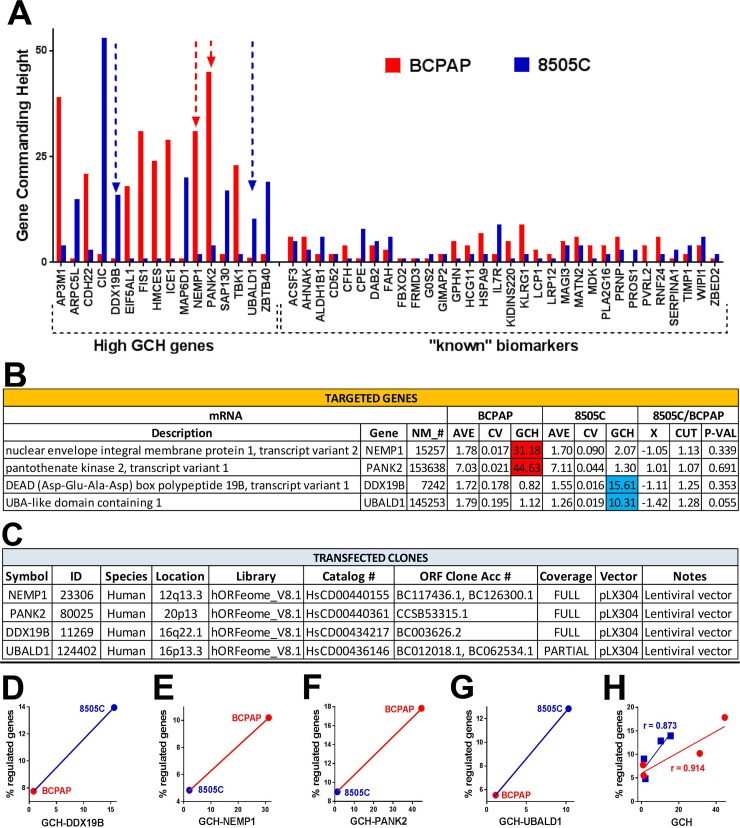
Validation of the gene commanding height (GCH) predictive value **(A)** Examples of genes (including known TC biomarkers) with large GCH differences in the two cell lines. **(B)** Average expression level (AVE), coefficient of variation (CV) and GCH of the selected genes in the two cell lines before any transfection. X = expression ratio, CUT = absolute fold-change cut-off for significantly differentially expressed genes, P-VAL = p-value of the differential expression. **(C)** Characteristics of the transfected clones. In all transfections, we used the same lentiviral vector pLX304. **(D-G)** GCH vs % regulated genes for each transfection experiment. Note that always, higher GCH is associated with larger percentage of regulated genes. **(H)** GCH increases with GCH in each cell type. Strong positive Pearson product-momentum correlations (r = 0.914 for BCPAP and r = 0.873 for 8505C cells) were found between the GCH scores and the percentage of significantly regulated genes in both cell lines.

We determined and compared the GCH scores of individual genes in the BCPAP and 8505C cell lines before any transfection (partially illustrated in Figure 4A). *DDX10, NEMP1, PANK2* and *UBALD1* were selected because of their availability (through Albert Einstein College of Medicine Genomics Facility), significantly different GCH-scores but close expression levels (AVE) and low coefficient of variation (CV) in the two cell lines (Figure 4B). The characteristics of the clones chosen to be stably transfected (one at a time) into the two types of cells are presented in Figure 4C. The microarray experiment validated (p-value = 0.000152) our hypothesis that manipulation of a gene's expression induced larger transcriptomic alterations in the cells where it has larger GCH (Figure 4D-4G). There is a perfect positive Spearman rank correlation between the (% regulated in BCPAP, % regulated in 8505C) and (GCH in BCPAP, GCH in 8505C) for all four transfected genes. Moreover, the strong positive Pearson product-momentum correlation with the percentage of regulated genes (Figure 4H) for each cell line validated the predictive value of GCH score.

## DISCUSSION

The main methodological contribution of our report is the introduction and validation of the Gene Commanding Height (GCH) as a new measure of how influential the expression of a gene is for the phenotypic expression of a cell. With this measure one can establish the gene hierarchy and identify the Gene Master Regulators (GMRs) of cancer nodules and unaffected surrounding tissue. Thus, GCH analysis opens a novel cancer gene-therapy avenue by selecting the targets with high differences in favor of the malign region from the affected tissue.

Owing to the cancer dependence on race, sex, age, genetic heritage, medical history, environmental and life-style associated risk factors, each patient most likely has a distinct and dynamic GMR-hierarchy. Therefore, an efficient gene-therapy should identify the targets separately for each individual. Manipulation of a GMR could *complement* current treatment options by making cancer cells more vulnerable and normal ones more resistant to chemo-/radiation therapy. It could also be used *post-surgical* removal of the tumor to reduce the probability of cancer recurrence.

GMRs are not biomarkers because biomarkers are the most alterable while GMRs are the most protected genes. GMRs are not selected as the most co-regulated with other genes in cancer vs. normal phenotypes because gene networks remodel in cancer and co-regulation does not necessarily mean gene interaction. Instead, GMRs are the most coordinately (synergistically or antagonistically) expressed with other genes in one phenotype at a time, dictating the transcriptomic stoichiometry [58] of gene networks.

In summary, we verified that cancer nodules and surrounding normal tissue are governed by different GMRs, and that manipulating the expression of a gene has larger effects on cells in which it has larger GCH.

## MATERIALS AND METHODS

### Patient sample

Four (2 – 6 mm^3^) samples were dissected from a frozen unilateral, single, 32.0 mm papillary carcinoma, pathological stage pT3NOMx (http://emedicine.medscape.com/article/2006643-overview) collected in 2010 from a 33y old woman. Four small pieces from the negative for malignancy resection margins of the same tumor were used as control. The study was approved by New York Medical College and Westchester Medical Center (WMC) Committees for Protection of Human Subjects, commonly known as Institutional Review Boards (IRBs) by L-11,606 (“Comprehensive molecular analysis of thyroid cancer: diagnosis, predictors of progression and targets for directed therapy”, PI RK Tiwari) and L-11,376 (“Quantifying cancer-associated remodeling of key genomic fabrics”, PI. DA Iacobas). The approval granted access to frozen cancer specimens from the WMC Pathology Archives and depersonalized pathology reports, waiving patient's informed consent. Although the four samples dissected from each (malignant and malignant-free) region were chosen to be as homogeneous as possible cells of different phenotypes were not completely eliminated.

### Cell lines

The predictive value of the GCH score was tested in papillary thyroid cancer cell line BCPAP and anaplastic thyroid cancer line 8505C, purchased from DSMZ in Braunschweig, Germany. Cell lines were cultured in Rosswell Park Memorial Institute (RPMI)-1640 supplemented with 10% fetal bovine serum (FBS), penicillin 10,000 IU/mL, streptomycin 10 mg/mL, and 2 mM L-glutamine. Validation of the cell lines was performed by the Genomics Core of the Albert Einstein College of Medicine (AECOM) of Yeshiva University (http://www.einstein.yu.edu/research/shared-facilities/cores/46/genomics/).

### Gene transfection

The stable transfection was performed using plasmids in ORF lentiviral plX304 vector produced by the AECOM shRNA Core Facility with the characteristics indicated in Figure 4C.

### Microarrays

We used our standard protocol [59] for extraction the total RNA, reverse transcription and fluorescent labeling, and hybridization with Agilent (here human) 4×44k two-color gene expression microarrays in the “multiple-yellow” design. Four biological replicas (cell culture dishes or tissue samples) were profiled from each phenotype/cell type subjected to each condition.

### Data analysis

A gene was considered as differentially expressed between two types of samples if the absolute expression fold-change exceeded the combined effect of microarray noise and biological variability and the p-value of the means’ equality was below 0.05. All genes were assigned to functional pathways using Kyoto Encyclopedia of Genes and Genomes (http://www.genome.jp/kegg/pathway.html).

In previous papers (e.g.: [38, 60, 61]) we have used the Relative Expression Variability (REV = median of the Bonferroni-corrected chi-square interval estimate of the coefficient of variation) as a statistical estimate of the expression variability of one gene among biological replicas. Expression of individual genes depends on local conditions that, although similar, are not identical among biological replicas. We assume that expression of key genes is kept by the cellular homeostatic mechanisms within narrow intervals while that of nonkey genes is less restrained to readily adapt to environmental changes. REV may shift in pathological conditions, suggesting that control mechanisms are also affected. The expression variability allows computing the Pearson product-momentum correlation coefficient between the expression levels of any two genes in the same condition. Using the coordination analysis, we determined the transcriptomic networks (that may cross cell boundaries as shown in [62])

### Gene commanding height

Gene Commanding Height (GCH) in sample α (= benign, malign, BCPAP, 8505C) was computed for each protein-coding and non-coding transcript in each of the four conditions as a combined measure of the gene expression stability and coordination with each other gene:

$$\begin{array}{l} GCH_{i}^{\left(\alpha \right)}=\exp(\underset{control}{\underset{︸}{WS_{i}^{\left(\alpha \right)}}}+\underset{coordination}{\underset{︸}{\frac{\bar{{\left(\rho_{ij}^{2}\right)}_{j\in M^{\left(\alpha \right)}}}}{\bar{{\left(\rho_{ij}^{2}\right)}_{i,j\in M^{\left(\alpha \right)}}}}}}-1),\;where:\;\\ WS_{i}^{\left(\alpha \right)}=\ln(\frac{\langle REV_{i}^{\left(\alpha \right)}\rangle }{REV_{i}^{\left(\alpha \right)}}),\;\langle REV_{i}^{\left(\alpha \right)}\rangle \;=\;median\;of\;the\;REVs\;\\ \;of\;all\;quantified\;genes\\ REV_{i}^{\left(\alpha \right)}(\varepsilon )=\frac{1}{2}CV_{i}^{\left(\alpha \right)}(\sqrt{\frac{4R_{i}^{\left(\alpha \right)}-1}{\chi^{2}(4R_{i}^{\left(\alpha \right)}-1;1-\varepsilon /2)}}+\sqrt{\frac{4R_{i}^{\left(\alpha \right)}-1}{\chi^{2}(4R_{i}^{\left(\alpha \right)}-1;\varepsilon /2)}}) \end{array}$$ (1)

## SUPPLEMENTARY MATERIALS FIGURES AND TABLES





## Footnotes

1 modified from map05216, Kanehisa Laboratories, http://www.kegg.jp
