# Supplementary material for: Gene master regulators of papillary and anaplastic thyroid cancers
Source: Oncotarget. 2017 Dec 19;9(2):2410–24. doi: 10.18632/oncotarget.23417 (PMC5788649; doi:10.18632/oncotarget.23417)
Supplement: Supplementary file 1 [file oncotarget-09-2410-s001.pdf]

# Gene master regulators of papillary and anaplastic thyroid cancers

## SUPPLEMENTARY MATERIALS

**Supplementary Table 1: The significantly regulated genes in the cancer nodule with respect to the surrounding normal tissue in the profiled papillary thyroid carcinoma.**

**See Supplementary File 1**
